# Integrating a Microbiology Laboratory Improves the Attitudes and Performance of First-Year Osteopathic Medical Students

**DOI:** 10.7759/cureus.90963

**Published:** 2025-08-25

**Authors:** Victoria L Hrach

**Affiliations:** 1 College of Medicine, Lake Erie College of Osteopathic Medicine, Greensburg, USA

**Keywords:** laboratory, medical education, microbiology, osteopathic medicine, problem-based learning

## Abstract

Microbiology is a vital component of preclinical education for allopathic and osteopathic medical schools. However, many medical students did not complete a microbiology course in previous undergraduate or graduate programs, making this their first introduction to the subject. Additionally, microbiology content in many medical schools is typically not supplemented by laboratory coursework, which would provide a hands-on learning modality. The goal of this project was to examine whether incorporating a microbiology laboratory activity into the first-year osteopathic medical curriculum results in altered attitudes toward and/or performance in microbiology. In Spring 2024 (n = 28) and Spring 2025 (n = 22), first-year osteopathic medical students (OMS1, n = 50) engaged in a two-session microbiology laboratory activity. This activity covered colony isolation, differential and selective media, Gram staining, catalase testing, and, in the case of the Spring 2025 section, antibiotic sensitivity. Students completed surveys comprised of multiple-choice knowledge questions and Likert scale opinion questions, both before and after the laboratory exercise. Paired t-tests were used to examine changes in responses from the presurvey to the postsurvey. Student performance on multiple-choice questions improved after completing the laboratory activity (for all participants, n = 50; p < 0.0001). Of these 50 students, 44 students’ individual performance improved, with only five students having no change in performance and one student performing worse. Regardless of whether students had taken a microbiology course or previously worked in a laboratory, performance improved (took microbiology, n = 27; p < 0.0001, did not take microbiology, n = 23; p < 0.0001, worked in a lab, n = 27; p < 0.0001, did not work in a lab, n = 22, p < 0.0001). As determined by unpaired t-tests, course readings completed between the two laboratory sessions did not influence postsurvey multiple-choice scores. Based on paired t-tests for all participants, student ratings significantly increased from the presurvey to the postsurvey for questions 1 (I enjoy microbiology; p < 0.0001; mean score improved from 5.20/7 to 5.92/7), 3 (I am confident about my knowledge of microbiology techniques; p < 0.0001; mean score improved from 3.32/7 to 4.90/7), 4 (I am confident that I understand how a sample obtained from a patient with a bacterial infection may be tested; p < 0.0001; mean score improved from 4.10/7 to 5.82/7), and 5 (I enjoy doing microbiology lab work; p < 0.0001; mean score improved from 4.70/7 to 6.32/7). In summary, independent of students' microbiology course experience or laboratory work experience, incorporation of a laboratory activity in the OMS1 curriculum provides a short-term improvement in student microbiology knowledge and increases positive sentiments toward laboratory work and microbiology. More opportunities should be taken to implement hands-on exercises in microbiology where possible within the medical curriculum. This work joins a limited but promising body of literature examining laboratory activities alongside medical coursework. Future studies should examine long-term content retention and attitudes several weeks or months after laboratory activities, as well as different modalities of administration (visual, verbal, etc.) for supplementary content.

## Introduction

Understanding microbes and their linkage to human health is an integral aspect of medicine. On a global scale, the rise of multidrug-resistant microbes and the need to develop vaccines in the wake of viral evolution are just two examples of major priorities for human health [1,2]. However, on a day-to-day basis, many physicians also encounter microbes in the context of diagnosing and treating infectious diseases. For instance, upper respiratory tract infections, pneumonia, and acute otitis media are three of the top 10 most globally treated conditions by primary care providers [3]. Additionally, pneumonia, urinary tract infections, and septicemia are among the top six causes for hospitalization in adults aged 85 and older [4]. Accordingly, microbiology is an integral component of preclinical education for medical students and is incorporated into the first-year curriculum, the second-year curriculum, or both [5].

For many medical students, medical school is their first introduction to microbiology. A 2018 review of data from the American Association of Colleges of Osteopathic Medicine found that only 17% of osteopathic medical students (OMS1; 1,931 of 11,890) had completed a microbiology course before matriculation [6]. Among this subgroup of 1,931 medical students, the majority (1,401; 72.6%) reported having taken only one microbiology course [6]. Additionally, the integration of laboratory-based coursework is historically undertaught [7,8].

Research regarding the efficacy of implementing laboratory activities into medical school curricula has been limited but promising. Students who completed a half-day clinical laboratory skill course performed better on interpretation of the included laboratory tests (Gram stain of blood, Gram stain of sputum, urinalysis, and peripheral blood smear) than students who did not complete the course [8]. Another study demonstrated that among 26 first-year medical students, those who completed an hour-long workshop where they prepared and interpreted Gram stains of bacteria performed better on multiple-choice questions about Gram staining than those who attended an hour-long discussion with a minimal lab demonstration or no outside-of-class microbiology activities at all [9]. However, these represent documented exceptions to the typical medical school curriculum, rather than the norm. Additionally, they did not examine other aspects of how laboratory activities may benefit students, such as whether these opportunities altered student attitudes or enthusiasm toward the covered topics. Lastly, while Gram staining is a key component of microbiology laboratory work, so are the utilization of microbiological growth media, the determination of appropriate antibiotic treatments, and the interpretation of the results of biochemical microbial testing. Thus, a more comprehensive exercise including these microbiology laboratory tests will provide students with a learning experience that is more representative of the clinical laboratory setting as a whole. Furthermore, the efficacy of implementing laboratory exercises specifically within problem-based learning (PBL) curriculum offers a novel area for exploration. In general, medical school curricula do not incorporate laboratory experiences that address the diagnosis of infectious diseases or allow students to have hands-on experiences with these tests [7,8].

At Lake Erie College of Osteopathic Medicine (LECOM)-Seton Hill, all medical students participate in the PBL pathway, where students work through patient cases together synchronously with faculty guidance and are responsible for reading related content in their own time, which then appears on quizzes and examinations [10]. Similar to the special study module (SSM) format employed within some medical curricula, in which students discuss material related to the core curriculum and enhance learning skills and interests, PBL offers a highly interactive small group setting [11]. However, the format of PBL meetings is different in that it mimics a patient-clinician interaction; PBL students play the role of medical providers who interview a "patient" (typically played by a faculty PBL facilitator), thereafter recommending appropriate medical tests and discussing basic science concepts related to a diagnosis [10].

Several cases in a microbiology PBL unit (spring of OMS1 year) at LECOM-Seton Hill incorporate principles and results of laboratory testing for patients with various infections. This has historically not been supplemented by hands-on activities, such as enabling students to conduct the tests themselves or practice interpreting them aside from images in PBL cases. From this, it is reasonable to hypothesize that the integration of a wet laboratory into the curriculum will increase student interest, understanding, and critical thinking. This report addresses this hypothesis by surveying student attitudes towards microbiology and laboratory work through Likert scale questions, and by surveying knowledge of microbiology techniques and relevant microbes via multiple-choice questions.

## Materials and methods

Research consent

As determined by the LECOM institutional review board (IRB), this research (protocol 31-118) was IRB exempt as “research using benign behavioral interventions.” Factors in design included to meet IRB exemption standards were anonymization of students, immediate disposal of the student identifier reference sheets when no longer needed, broad background questions, and no expectation of questions eliciting distress. Additionally, all participants were adults, and participation in surveys was entirely voluntary. Participants verbally consented to participate in the survey component of this activity to the microbiology faculty members who supervised this lab exercise. Additionally, the presurvey and postsurvey (Appendix 1) reminded students of the voluntary nature of the activity and that they may withdraw from participation at any time.

Study funding

This study was not funded by any internal or external grants. Lake Erie College of Osteopathic Medicine provided all supplies for this line of investigation.

Setting and participants

This activity took place twice during the spring OMS1 microbiology unit: once in April 2024 and once in April 2025. Participants were recruited by emailing PBL students and offering half-hour (2024) or hour (2025) timeslots to attend the laboratory on each of two days. In Spring 2024, of 112 OMS1 students at LECOM-Seton Hill, 28 (25% of eligible participants) attended both sessions and opted into the educational research component (survey completion). In Spring 2025, 22 of 105 OMS1 students (21% of eligible participants) participated as described previously.

Intervention

Students signed up for “student identifier” numbers to anonymize survey results and completed the presurvey (Appendix 1). The intervention then took place across two sessions. Students completed the Session 1 activities outlined in an activity handout, with the handout and verbal instruction from microbiology faculty as assistance. Session 1 focused on learning how to isolate colonies of bacteria on solid growth media. To do this, students used pre-prepared bacterial cultures of *Staphylococcus aureus*, a Gram-positive coccus associated with toxic shock syndrome [12] and staphylococcal scalded skin syndrome [13], and *Escherichia coli*, a Gram-negative rod associated with urinary tract infections [14] and diarrhea [15]. Students were not initially told the identities of the microbes in the culture.

**Figure 1:**
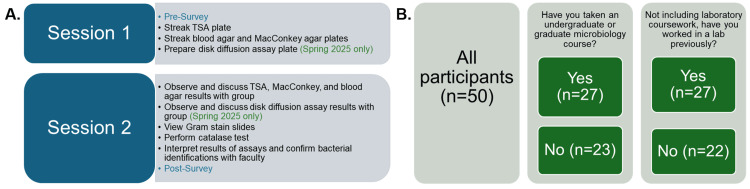
Overview of the activity and respondents (A) Layout of the two lab sessions and content covered therein. (B) Participants and groupings based on their respective educational and work backgrounds TSA: tryptic soy agar

As depicted in Figure 1A, during the first session, each student streaked the culture onto plates. The first of these was a generalized growth medium, tryptic soy agar, as a practice exercise and so that bacterial colony morphology could be observed [16]. Second, each student utilized a blood agar plate, used to identify how bacteria break down red blood cells [17], and MacConkey agar, which selects for Gram-negative enteric bacteria and indicates whether they ferment lactose [18]. Importantly, these media types are used to identify bacteria in a clinical setting [19]. For this group of students at LECOM-Seton Hill, both MacConkey agar and blood agar had previously been discussed in PBL group sessions as well as the microbiology textbook [19]. In Spring 2025, antibiotic sensitivity testing using the disk diffusion (Kirby-Bauer) method was also used.

Students returned several days later for Session 2 (Figure 1B), where microbiology faculty provided them with their TSA, blood agar, MacConkey agar, and/or (Spring 2025 only) disk diffusion plates, on which bacteria had grown. Students also performed a catalase test to examine if their bacteria can break down hydrogen peroxide; importantly, this test is used to differentiate between staphylococci and streptococci, both Gram-positive cocci [19]. Students were also provided with pre-prepared Gram stain slides of the bacteria that they had worked with, which they visualized via microscopy. Microbiology faculty confirmed the identities of the microbes that students were testing as *E. coli* or *S. aureus*. As with media types, both Gram staining and catalase testing had been introduced in PBL microbiology work and the microbiology textbook [19].

At the culmination of this activity, students completed the postsurvey (Appendix 2). The document with student identifiers for student reference was shredded immediately after the end of the intervention. It should be noted that both the presurvey and postsurvey are novel instruments created for this study. All data collected were those directly addressed in the presurvey and postsurvey. Analyses were performed using the overall group and four subgroups based on whether students had taken a microbiology course before (yes/no) or had worked in a laboratory before (yes/no).

Outcomes measured and analysis

The presurvey and postsurvey (Appendix 1) asked students nine (Spring 2024) or eleven (Spring 2025) multiple-choice microbiology questions regarding content covered in the laboratory activity (media types, catalase test, Gram staining, and colony isolation). The outcomes assessed were students’ total performance on multiple-choice questions (as percentage correct so that 2024 and 2025 findings could be combined), and ratings (on a 1-7 Likert scale) of opinion statements regarding microbiology. All statistical analyses were performed in GraphPad Prism^TM^ (Dotmatics, Boston, MA). The survey and laboratory activity were both developed with assistance from educational and scientific experts, as acknowledged.

For the multiple-choice section of the surveys, analyses were performed for the total group (n = 50) and several subgroups using each student’s percent correct answers (0-100). Subgroups included students who had taken a microbiology course (n = 27) or not (n = 23) and students who had worked in a lab in an extracurricular capacity (n = 27) or not (n = 22). It should be noted that unclear marking resulted in one student’s survey responses not being included in the analysis for either “worked in a lab” subgroup. For all participants and for each of the four subgroups, comparisons were made between students’ percent correct answers on the presurvey vs. on the postsurvey using paired t-tests. Additionally, unpaired t-tests were used to assess whether students having read microbiology content between Sessions 1 and 2 impacted multiple-choice question performance on the postsurvey.

For each of the opinion statements within the surveys, student ratings from 1-7 (1 = strongly disagree, 7 = strongly agree) were compared. To analyze changes in rating for each statement from the presurvey to the postsurvey, paired t-test comparisons were performed.

## Results

Completing the microbiology laboratory exercise improves student performance on multiple-choice questions

No analysis was performed for students who attended the first lab session alone (n = 75; Table 1), as they did not complete the postsurvey offered in the second session. This would not have allowed for an accurate assessment of performance improvement resulting from the full intervention (both sessions of the lab activity).

**Table 1:** Presurvey results for all students who attended session 1 of the lab activity Year, student identifier (three-digit number), responses about having taken microbiology courses or having worked in a lab, multiple-choice % performance, and Likert scale ratings of all participants who attended session 1 of the lab activity (n = 75) are shown. Some of these students did not attend session 2 to review the results of tests, further engage in laboratory learning, and complete the postsurvey; thus, the nonreturning students were excluded from all analyses

Year	Student identifier	Have you taken micro?	Have you worked in a lab?	Multiple-choice (% correct)	Likert Q1	Likert Q2	Likert Q3	Likert Q4	Likert Q5
2024	001	Yes	Yes	29	6	7	4	5	6
2024	002	No	Yes	33	4	6	5	6	5
2024	003	Yes	No	22	5	6	3	4	5
2024	004	No	No	67	4	5	2	3	5
2024	005	Yes	Yes	44	5	7	3	3	6
2024	006	Yes	Yes	67	6	7	3	3	4
2024	007	Yes	No	67	5	6	5	4	4
2024	008	No	Yes	56	6	6	4	2	4
2024	009	No	Yes	22	5	6	1	6	4
2024	010	No	No	67	6	7	2	5	4
2024	011	No	Yes	56	4	7	2	4	4
2024	012	Yes	Yes	67	6	7	5	5	6
2024	013	Yes	No	56	5	7	5	5	5
2024	014	Yes	No	56	6	5	4	5	6
2024	015	No	No	67	5	7	3	4	6
2024	016	No	Yes	67	4	5	4	4	4
2024	017	No	Yes	78	6	5	3	1	3
2024	018	Yes	Yes	89	3	7	4	3	4
2024	019	Yes	Yes	89	6	5	6	6	6
2024	020	No	No	78	5	7	2	3	4
2024	021	No	No	100	5	7	2	4	4
2024	022	Yes	Yes	67	5	7	4	6	6
2024	023	No	Answer unclear (student circled both yes and no)	44	4	6	2	3	2
2024	024	No	Yes	44	4	7	5	5	4
2024	025	No	Yes	67	7	7	3	6	3
2024	026	No	Yes	78	6	7	4	5	4
2024	027	No	Yes	11	5	7	4	6	7
2024	028	Yes	Yes	67	7	6	4	5	7
2024	029	Yes	Yes	67	7	7	7	7	7
2024	030	No	Yes	56	5	4	3	5	6
2024	031	No	No	44	7	7	1	4	4
2024	032	Yes	Yes	56	6	6	5	5	5
2024	033	Yes	No	67	7	7	2	2	4
2024	034	No	No	78	5	4	2	5	4
2024	035	No	No	56	4	6	2	2	
2024	036	Yes	Yes	56	5	7	3	5	4
2024	037	Yes	Yes	44	6	6	4	4	4
2024	038	Yes	No	100	4	5	2	4	5
2024	039	Yes	Yes	78	4	4	5	3	4
2024	040	No	Yes	44	5	6	4	4	6
2024	041	No	Yes	56	5	6	3	3	3
2024	042	Yes	No	78	4	6	3	6	6
2024	043	Yes	No	78	5	7	4	7	5
2024	045	No	Yes	56	6	6	5	7	7
2024	046	Yes	Yes	44	5	6	5	5	6
2024	047	No	No	44	5	6	3	4	3
2025	001	Yes	No	18	4	3	1	1	4
2025	002	Yes	Yes	73	6	5	5	6	6
2025	003	Yes	No	55	2	5	1	5	1
2025	004	Yes	Yes	82	7	7	5	2	7
2025	005	Yes	No	36	5	5	3	2	6
2025	006	Yes	Yes	55	5	6	3	3	5
2025	007	Yes	Yes	64	5	5	4	5	5
2025	008	No	Yes	36	4	5	3	4	4
2025	009	Yes	No	45	5	6	2	5	4
2025	010	Yes	No	64	7	7	5	4	7
2025	011	Yes	Yes	64	5	5	3	3	4
2025	012	No	Yes	73	4	6	2	4	5
2025	013	Yes	No	82	7	7	5	5	7
2025	014	Yes	No	55	7	7	5	5	7
2025	015	Yes	Yes	40	5	6	2	2	2
2025	016	No	No	36	5	7	2	2	2
2025	017	No	Yes	55	5	5	4	6	5
2025	018	No	Yes	73	5	7	4	4	4
2025	019	Yes	Yes	55	3	6	4	4	4
2025	020	No	Yes	27	4	7	1	1	6
2025	021	Yes	No	18	4	4	3	4	5
2025	022	Yes	Yes	27	5	5	3	5	5
2025	023	No	No	27	5	6	1	1	1
2025	024	Yes	No	18	5	5	5	5	5
2025	025	No	No	64	2	4	1	1	4
2025	026	Yes	Yes	45	7	7	5	6	6
2025	027	Yes	Yes	45	7	7	2	2	7
2025	029	Yes	Yes	27	4	6	3	3	6
2025	"29"	Yes	No	36	4	2	6	6	4

**Figure 2:**
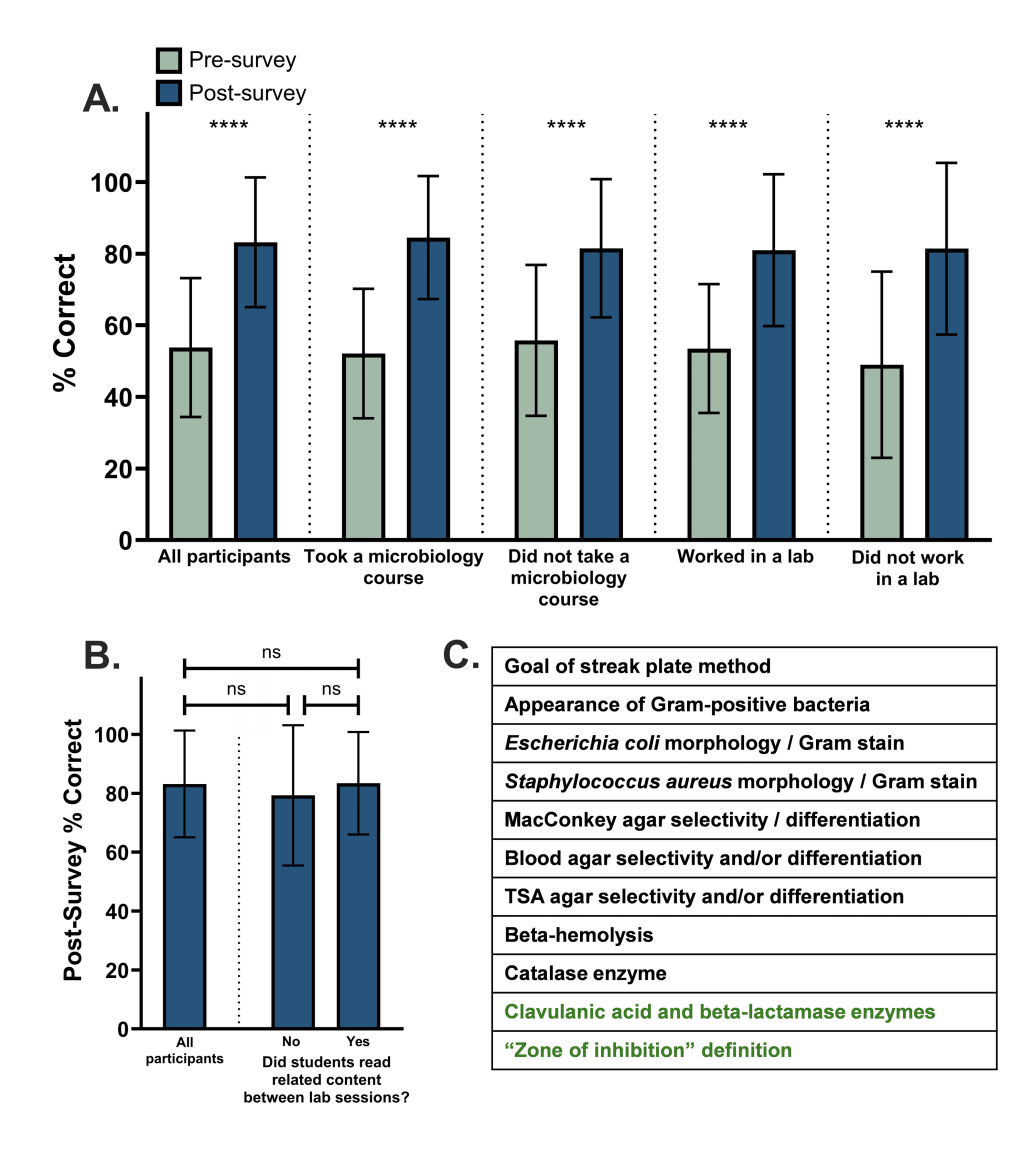
Completing the microbiology laboratory exercise improves student performance on multiple-choice questions (A) Percent correct multiple-choice questions on pre- and postsurveys across various groups of students. Paired t-tests were used to evaluate significance. The values are calculated as mean ± SD. ^****^p < 0.0001. (B) Impact of out-of-class readings on postsurvey performance. Unpaired t-tests were used to evaluate significance. The values are calculated as mean ± SD. (C) Subjects of the various multiple-choice questions. Topics in green text denote new questions for Spring 2025 only. All other questions (black text) were employed in both Spring 2024 and Spring 2025 ns: not significant; TSA: tryptic soy agar

Figure 2A depicts the mean ± SD of students’ percent correct of multiple-choice questions in the survey. Based on paired t-tests, statistically significant pre- to postsurvey improvement, where p < 0.0001 (^****^), occurred within the total group. Improvement also occurred within each of the four defined subgroups: those who had taken a microbiology course (p < 0.0001 indicated by ^****^), those who did not take a microbiology course (p < 0.0001 indicated by ^****^), those who worked in a laboratory (p < 0.0001 indicated by ^****^), and those who did not work in a laboratory (p < 0.0001 indicated by ^****^). Mean scores improved from 53.8% to 83.2% for all participants who attended both laboratory sessions (n = 50), from 52.1% to 84.5% for those who had taken a microbiology course (n = 27), from 55.8% to 81.5% for those who had not taken a microbiology course (n = 23), from 53.5% to 81.0% for those who had worked in a laboratory (n = 27), and from 49.0% to 81.4% for those who had not worked in a laboratory (n = 22).

Furthermore, most (44 of 50) students’ scores improved from the presurvey to the postsurvey, with five students’ scores remaining the same and one decreasing (Tables 2, 3).

**Table 2:** Presurvey results for students who attended both sessions of the lab activity Year, student identifier (three-digit number), responses about having taken microbiology courses or having worked in a lab, multiple-choice % performance, and Likert scale ratings of participants who attended both sessions of the lab activity (n = 50) are shown. All data included in this table (presurvey responses) were utilized in graphing and analyses

Year	Student identifier	Have you taken micro?	Have you worked in a lab?	Multiple-choice (% correct)	Likert Q1	Likert Q2	Likert Q3	Likert Q4	Likert Q5
2024	001	Yes	Yes	29	6	7	4	5	6
2024	002	No	Yes	33	4	6	5	6	5
2024	003	Yes	No	22	5	6	3	4	5
2024	004	No	No	67	4	5	2	3	5
2024	005	Yes	Yes	44	5	7	3	3	6
2024	006	Yes	Yes	67	6	7	3	3	4
2024	007	Yes	No	67	5	6	5	4	4
2024	008	No	Yes	56	6	6	4	2	4
2024	010	No	No	67	6	7	2	5	4
2024	011	No	Yes	56	4	7	2	4	4
2024	012	Yes	Yes	67	6	7	5	5	6
2024	013	Yes	No	56	5	7	5	5	5
2024	014	Yes	No	56	6	5	4	5	6
2024	015	No	No	67	5	7	3	4	6
2024	016	No	Yes	67	4	5	4	4	4
2024	020	No	No	78	5	7	2	3	4
2024	021	No	No	100	5	7	2	4	4
2024	022	Yes	Yes	67	5	7	4	6	6
2024	023	No	Answer unclear (student circled both yes and no)	44	4	6	2	3	2
2024	024	No	Yes	44	4	7	5	5	4
2024	025	No	Yes	67	7	7	3	6	3
2024	026	No	Yes	78	6	7	4	5	4
2024	027	No	Yes	11	5	7	4	6	7
2024	030	No	Yes	56	5	4	3	5	6
2024	031	No	No	44	7	7	1	4	4
2024	032	Yes	Yes	56	6	6	5	5	5
2024	033	Yes	No	67	7	7	2	2	4
2024	034	No	No	78	5	4	2	5	4
2025	002	Yes	Yes	73	6	5	5	6	6
2025	003	Yes	No	55	2	5	1	5	1
2025	004	Yes	Yes	82	7	7	5	2	7
2025	005	Yes	No	36	5	5	3	2	6
2025	006	Yes	Yes	55	5	6	3	3	5
2025	007	Yes	Yes	64	5	5	4	5	5
2025	008	No	Yes	36	4	5	3	4	4
2025	009	Yes	No	45	5	6	2	5	4
2025	010	Yes	No	64	7	7	5	4	7
2025	012	No	Yes	73	4	6	2	4	5
2025	013	Yes	No	82	7	7	5	5	7
2025	014	Yes	No	55	7	7	5	5	7
2025	015	Yes	Yes	40	5	6	2	2	2
2025	016	No	No	36	5	7	2	2	2
2025	018	No	Yes	73	5	7	4	4	4
2025	019	Yes	Yes	55	3	6	4	4	4
2025	020	No	Yes	27	4	7	1	1	6
2025	021	Yes	No	18	4	4	3	4	5
2025	022	Yes	Yes	27	5	5	3	5	5
2025	023	No	No	27	5	6	1	1	1
2025	024	Yes	No	18	5	5	5	5	5
2025	026	Yes	Yes	45	7	7	5	6	6

**Table 3:** Postsurvey results for students who attended both sessions of the lab activity Year, student identifier (three-digit number), responses about having taken microbiology courses or having worked in a lab, multiple-choice % performance, and Likert scale ratings of participants who attended both sessions of the lab activity (n = 50). All data included in this table (postsurvey responses) were utilized in graphing and analyses

Year	Student identifier	Read material between sessions?	Multiple-choice (% correct)	% Performance vs. presurvey score	Likert Q1	Likert Q2	Likert Q3	Likert Q4	Likert Q5
2024	001	Yes, unrelated	33	150	6	6	5	6	6
2024	002	No	44	133	6	7	6	7	7
2024	003	Yes, related	100	450	5	6	5	5	5
2024	004	Yes, unrelated	100	150	6	5	6	6	6
2024	005	Yes, related	78	175	6	7	4	6	6
2024	006	Yes, unrelated	100	150	6	7	5	7	6
2024	007	Yes, related	100	150	5	6	5	6	4
2024	008	Yes, unrelated	89	160	5	6	3	7	5
2024	010	Yes, unrelated	89	133	6	7	4	5	6
2024	011	Yes, related	100	180	5	7	4	5	6
2024	012	No	100	150	7	7	6	6	7
2024	013	No	89	160	6	6	6	6	6
2024	014	Yes, related	89	160	6	5	6	6	6
2024	015	Yes, unrelated	78	117	6	6	5	4	5
2024	016	Yes, related	89	133	4	4	4	4	4
2024	020	Yes, unrelated	100	129	6	7	5	7	6
2024	021	Yes, unrelated	100	100	5	6	2	6	5
2024	022	Yes, unrelated	89	133	6	7	5	6	6
2024	023	Yes, unrelated	78	175	4	4	2	3	3
2024	024	Yes, unrelated	100	225	5	7	5	6	5
2024	025	Yes, unrelated	67	100	7	7	4	5	7
2024	026	Yes, unrelated	100	129	6	7	5	6	5
2024	027	Yes, unrelated	67	600	6	6	5	6	6
2024	030	Yes, related	78	140	6	6	5	6	6
2024	031	Yes, unrelated	44	100	7	7	5	6	7
2024	032	Yes, related	89	160	7	7	7	7	7
2024	033	Yes, related	89	133	7	7	5	7	7
2024	034	Yes, unrelated	89	114	5	4	2	4	5
2025	002	Yes, related	91	125	6	5	5	7	6
2025	003	Yes, related	64	117	5	5	5	5	5
2025	004	Yes, unrelated	82	100	7	7	7	7	7
2025	005	Yes, related	91	250	6	6	5	5	6
2025	006	Yes, related	36	67	5	6	5	6	7
2025	007	Yes, related	91	143	6	6	6	6	5
2025	008	Yes, related	36	100	6	6	5	6	6
2025	009	Yes, related	82	180	5	6	4	5	5
2025	010	Yes, unrelated	100	157	7	7	4	7	7
2025	012	Yes, related	91	125	6	6	4	6	5
2025	013	Yes, related	91	111	7	7	5	7	7
2025	014	Yes, unrelated	82	150	7	7	6	6	6
2025	015	Yes, related	91	250	6	6	5	5	6
2025	016	Yes, related	73	200	6	7	5	6	6
2025	018	Yes, related	91	125	7	7	6	7	6
2025	019	Yes, related	64	117	5	5	5	5	5
2025	020	Yes, related	100	367	6	5	5	6	7
2025	021	Yes, related	82	450	6	6	5	5	6
2025	022	Yes, related	100	367	6	6	5	6	5
2025	023	Yes, unrelated	73	267	7	7	7	7	7
2025	024	Yes, unrelated	91	500	5	5	5	5	5
2025	026	Yes, unrelated	91	200	7	7	5	5	6

Out-of-class readings do not impact multiple-choice performance

As discussed previously, student scores on multiple-choice questions increased after the microbiology laboratory activity. However, because of the several-day gap between Sessions 1 and 2 of the exercise, it came into question whether students completing microbiology readings between sessions may also impact their performance on multiple-choice questions. To account for the impact of course readings, unpaired t-tests were used to compare the postsurvey scores between the total group who attended both sessions (n = 50), students who read microbiology content related to the laboratory activities between Sessions 1 and 2 (n = 25), and those who did not read related content between Sessions 1 and 2 (n = 25, defined as those who did not read microbiology content at all, n = 3, or who read microbiology content unrelated to the lab activity topics) (Figure 2B). No significant differences were found between these groups (all students vs. those who read related content: p = 0.9637; all students vs. those who did not read related content: p = 0.4321; students who read related content vs. those who did not: p = 0.4902).

Completing the microbiology laboratory exercise improves several aspects of attitudes toward microbiology

**Figure 3:**
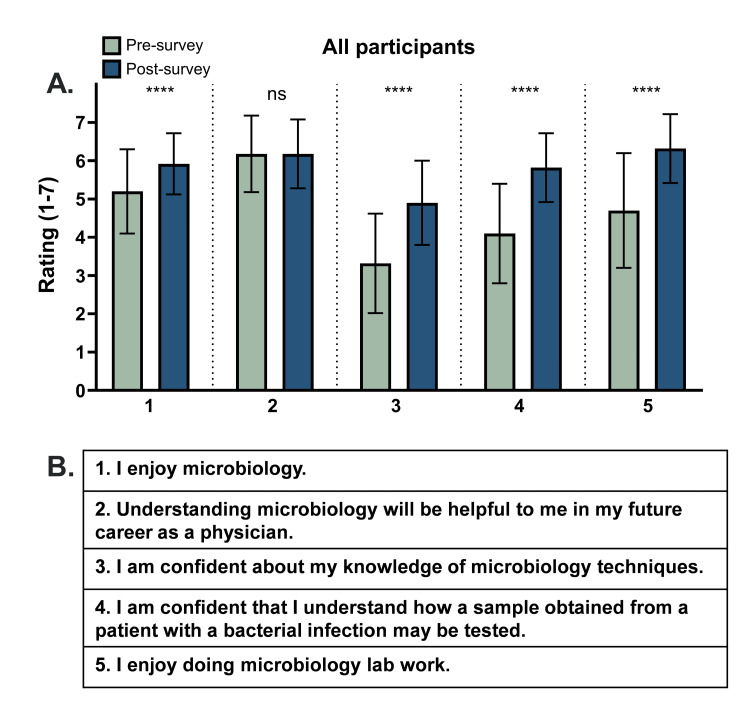
Completing the microbiology laboratory exercise improves several aspects of attitudes toward microbiology (A) Average ratings of each statement on a Likert scale from 1 to 7 among all participants in the presurvey and postsurvey. Paired t-tests were used to evaluate significance. The values are calculated as mean ± SD. ^****^p < 0.0001. (B) List of statements that students were asked to rate ns: not significant

Responses for all 50 participants involved in both lab sessions (Figure 3) were analyzed for each of the 1-7 Likert scale opinion prompts. For all participants, student ratings significantly increased from the presurvey to the postsurvey for questions 1 (I enjoy microbiology; p < 0.0001; mean score improved from 5.20/7 to 5.92/7), 3 (I am confident about my knowledge of microbiology techniques; p < 0.0001; mean score improved from 3.32/7 to 4.90/7), 4 (I am confident that I understand how a sample obtained from a patient with a bacterial infection may be tested; p < 0.0001; mean score improved from 4.10/7 to 5.82/7), and 5 (I enjoy doing microbiology lab work; p < 0.0001; mean score improved from 4.70/7 to 6.32/7). Regarding Likert scale question 2 (Understanding microbiology will be helpful to me in my future career as a physician), no significant difference from presurvey to postsurvey was found (ns, p > 0.9999). However, it should be noted that presurvey student ratings for this question were already quite high. Among all participants, the mean presurvey rating for question 2 was 6.18 ± 1.0, with the mean postsurvey rating being 6.18 ± 0.9. No analysis was performed for students who attended the first lab session alone.

## Discussion

The results of this study are consistent with the hypotheses that educational interventions through laboratory exercises would result in improved content knowledge and outlooks, as demonstrated through multiple-choice questions and Likert scale attitudinal questions.

Student performance on multiple-choice questions increased significantly after completing the microbiology laboratory activity; all but one of 50 students had either no change in score or a positive change in score (improvement) from the presurvey to the postsurvey. The laboratory activity's impact on student performance, rather than that of microbiology readings between Sessions 1 and 2, was confirmed by an analysis of postsurvey scores. This finding indicates that the laboratory exercise itself, and not readings independently completed by students in the two days between sessions, was responsible for the increase in multiple-choice performance. In summary, this improvement in multiple-choice question performance due to completion of the laboratory activity was consistent not only with the initial hypothesis but also with the findings of similar studies [8,9]. Unlike other studies, the surveys employed here used a different assortment of subject matter (Gram staining, catalase test, differential/selective media, and antibiotic sensitivity). Additionally, this microbiology exercise specifically supplemented the PBL curriculum for OMS1.

This study is also unique in that attitudinal effects of the laboratory activity were assessed. Of the five statements to which students indicated their agreement on a 1-7 Likert scale, completing the laboratory activity led to increased ratings of four of these statements. Importantly, for the statement where no pre- to postsurvey difference was seen, the presurvey rating was already high (mean 6.18/7). These findings are unsurprising given that utilization of highly interactive and engaging student-focused activities in medical education is associated with positive student attitudes and successful learning, as illustrated through previous work using the SSM modality [11].

A limitation of this study was that long-term learning outcomes were not addressed. In the future, the postsurvey could be administered later relative to the lab activity's completion. For example, if students completed post-surveys several weeks after the activity, this would mimic the need to recall information when taking a course exam. Furthermore, a control group of students who received no intervention in any format could also be added. Based on the surveys involving self-reporting, it is also possible that bias or error may be introduced.

This work also did not examine whether altered levels of involvement in the laboratory activity impacted performance; for example, in a previous study, two different interventions (one being a discussion group with a very brief pre-prepared laboratory component and the other a fully hands-on laboratory exercise) were used [9]. In addition to measuring the impact of different levels of involvement on students, varied versions of content delivery could also be utilized. For example, alternatives to the hands-on laboratory activity (which would likely appeal most to kinesthetic learners) could include a PowerPoint (Microsoft Corporation, Redmond, WA) lecture with many graphics to appeal to visual learners, and a faculty-student discussion with no supplementary images to appeal to auditory learners [20]. This could elucidate whether the laboratory format used here, or merely the opportunity to have additional out-of-class sessions with faculty, is more impactful on attitudes and on multiple-choice question performance. This would place the work alongside similar literature examining the linkage between learning style and academic success [20].

## Conclusions

Participation in a laboratory activity provides a short-term improvement in OMS1 students’ performance on multiple-choice microbiology questions. Participation in this activity also improves several aspects of student attitudes related to microbiology. Future studies should explore whether the positive impacts seen here also apply to long-term attitudes and content retention. While limited, the findings from this brief and engaging activity are promising and evidence a potential method to benefit students’ learning and attitudes. Medical schools may consider implementing hands-on microbiology activities where possible. Additional supplemental activities appealing to diverse learning styles (visual, auditory, etc.) may also be helpful to further reinforce concepts learned in coursework.

## Appendices

Appendix 1

The presurvey details are listed in the following table.

**Table 4:** Presurvey All contents of this table represent the exact contents of the presurvey as provided to the students. Multiple-choice questions 10/11 were unique to the Spring 2025 activity. All other questions were employed in both Spring 2024 and Spring 2025 activities. For section B (knowledge of concepts), an asterisk denotes the correct answer for each question PBL: problem-based learning; N/A: not available

Completing this survey is entirely voluntary. You may decide that you would not like to complete this survey at any time with no consequence							
Basic information (section A)							
1. Are you 18 years of age or older?	No	Yes	-	-	-	-	-
2. Student identifier	N/A, open-ended	-	-	-	-	-	-
3. Have you taken an undergraduate or graduate microbiology course?	No	Yes	-	-	-	-	-
4. Not including laboratory coursework, have you worked in a lab previously (i.e., employment or extracurricular research)?	No	Yes	-	-	-	-	-
Knowledge of concepts (section B). Your performance in this section will not impact your grade in the PBL course or any other course. These questions are intended to assess your knowledge of microbiology contents before and after participating in the lab activity							
1. The goal of the streak plate method is to:	Generate as much bacterial growth as possible	Evaluate antibiotic resistance of bacteria	Isolate single colonies of bacteria^*^	-	-	-	-
2. Bacteria appear different in a Gram stain based on the presence of peptidoglycan, with Gram-positive bacteria having a thick layer of peptidoglycan and staining ________	Pink	Purple^*^	Red	Blue	Colorless	-	-
3. *Escherichia coli* are ________	Gram-negative cocci	Gram-negative bacilli^*^	Gram-positive cocci	Gram-positive bacilli	-	-	-
4. *Staphylococcus aureus* are ________	Gram-negative cocci	Gram-negative bacilli	Gram-positive cocci^*^	Gram-positive bacilli	-	-	-
5. MacConkey agar is:	Selective but not differential	Differential but not selective	Both selective and differential^*^	Neither selective nor differential	-	-	-
6. Blood agar is:	Selective but not differential	Differential but not selective^*^	Both selective and differential	Neither selective nor differential	-	-	-
7. TSA agar is:	Selective but not differential	Differential but not selective	Both selective and differential	Neither selective nor differential^*^	-	-	-
8. Bacteria that fully break down blood in a blood agar plate are termed:	Alpha-hemolytic	Beta-hemolytic^*^	Gamma-hemolytic	-	-	-	-
9. Catalase is an enzyme that breaks down ________	Ethanol	Glucose	Hydrogen peroxide^*^	Water	-	-	-
10. Clavulanic acid inhibits ________ made by bacteria, which confer resistance to certain antibiotics	Beta-lactamase enzymes^*^	Aminoglycoside-modifying enzymes	Efflux pumps	-	-	-	-
11. Which of the following describes a zone of inhibition on an agar plate?	An area around an antibiotic disk where bacteria grow, indicating drug sensitivity	An area around an antibiotic disk where bacteria grow, indicating drug resistance	An area around an antibiotic disk where bacteria do not grow, indicating drug sensitivity^*^	An area around an antibiotic disk where bacteria do not grow, indicating drug resistance	-	-	-
Attitude/opinions (section C). For each question, indicate your level of agreement on the following scale: 1 = strongly disagree, 2 = disagree, 3 = slightly disagree, 4 = neither agree nor disagree, 5 = slightly agree, 6 = agree, 7 = strongly agree							
1. I enjoy microbiology	1	2	3	4	5	6	7
2. Understanding microbiology will be helpful to me in my future career as a physician	1	2	3	4	5	6	7
3. I am confident about my knowledge of microbiology techniques	1	2	3	4	5	6	7
4. I am confident that I understand how a sample obtained from a patient with a bacterial infection may be tested	1	2	3	4	5	6	7
5. I enjoy doing microbiology lab work	1	2	3	4	5	6	7
6 (Optional) Please provide any additional comments you would like to share about microbiology lab work or microbiology as a whole	N/A, open-ended	-	-	-	-	-	-

Appendix 2

The postsurvey details are listed in the following table.

**Table 5:** Postsurvey All contents of this table represent the exact contents of the postsurvey as provided to the students. Multiple-choice questions 10/11 were unique to the Spring 2025 activity. All other questions were employed in both Spring 2024 and Spring 2025 activities. For section A (basic information), “antibiotic sensitivity” appeared only in the Spring 2025 version of question 3; otherwise, the question text was the same across both years. For section B (knowledge of concepts), an asterisk denotes the correct answer for each question PBL: problem-based learning; TSA: tryptic soy agar; N/A: not available

Completing this survey is entirely voluntary. You may decide that you would not like to complete this survey at any time with no consequence							
Basic information (section A)							
1. Are you 18 years of age or older?	No	Yes	-	-	-	-	-
2. Student identifier:	N/A, open-ended.	-	-	-	-	-	-
3. Did you read any microbiology content between session 1 and session 2 of this activity?	No	Yes, but not directly related to the topics covered here (Gram staining, selective/differential agar, assessing antibiotic sensitivity, *Escherichia coli*, or *Staphylococcus aureus*)	Yes, including one or more topics here (Gram staining, selective/differential agar, assessing antibiotic sensitivity, *Escherichia coli*, or *Staphylococcus aureus*)	-	-	-	-
Knowledge of concepts (section B). Your performance in this section will not impact your grade in the PBL course or any other course. These questions are intended to assess your knowledge of microbiology contents before and after participating in the lab activity							
1. The goal of the streak plate method is to:	Generate as much bacterial growth as possible	Evaluate antibiotic resistance of bacteria	Isolate single colonies of bacteria^*^	-	-	-	-
2. Bacteria appear different in a Gram stain based on the presence of peptidoglycan, with Gram-positive bacteria having a thick layer of peptidoglycan and staining ________	Pink	Purple^*^	Red	Blue	Colorless	-	-
3. *Escherichia coli* are ________	Gram-negative cocci	Gram-negative bacilli^*^	Gram-positive cocci	Gram-positive bacilli	-	-	-
4. *Staphylococcus aureus* are ________	Gram-negative cocci	Gram-negative bacilli	Gram-positive cocci^*^	Gram-positive bacilli	-	-	-
5. MacConkey agar is:	Selective but not differential	Differential but not selective	Both selective and differential^*^	Neither selective nor differential	-	-	-
6. Blood agar is:	Selective but not differential	Differential but not selective *	Both selective and differential	Neither selective nor differential	-	-	-
7. TSA agar is:	Selective but not differential	Differential but not selective	Both selective and differential	Neither selective nor differential^*^	-	-	-
8. Bacteria that fully break down blood in a blood agar plate are termed:	Alpha-hemolytic	Beta-hemolytic^*^	Gamma-hemolytic	-	-	-	-
9. Catalase is an enzyme that breaks down ________	Ethanol	Glucose	Hydrogen peroxide^*^	Water	-	-	-
10. Clavulanic acid inhibits ________ made by bacteria, which confer resistance to certain antibiotics	Beta-lactamase enzymes^*^	Aminoglycoside-modifying enzymes	Efflux pumps	-	-	-	-
11. Which of the following describes a zone of inhibition on an agar plate?	An area around an antibiotic disk where bacteria grow, indicating drug sensitivity	An area around an antibiotic disk where bacteria grow, indicating drug resistance	An area around an antibiotic disk where bacteria do not grow, indicating drug sensitivity^*^	An area around an antibiotic disk where bacteria do not grow, indicating drug resistance	-	-	-
Attitude/opinions (section C). For each question, indicate your level of agreement on the following scale: 1 = strongly disagree, 2 = disagree, 3 = slightly disagree, 4 = neither agree nor disagree, 5 = slightly agree, 6 = agree, 7 = strongly agree							
1. I enjoy microbiology	1	2	3	4	5	6	7
2. Understanding microbiology will be helpful to me in my future career as a physician	1	2	3	4	5	6	7
3. I am confident about my knowledge of microbiology techniques	1	2	3	4	5	6	7
4. I am confident that I understand how a sample obtained from a patient with a bacterial infection may be tested	1	2	3	4	5	6	7
5. I enjoy doing microbiology lab work	1	2	3	4	5	6	7
6 (Optional). Please provide any additional comments you would like to share about microbiology lab work or microbiology as a whole	N/A, open-ended	-	-	-	-	-	-
